# Automated detection of missteps during community ambulation in patients with Parkinson’s disease: a new approach for quantifying fall risk in the community setting

**DOI:** 10.1186/1743-0003-11-48

**Published:** 2014-04-03

**Authors:** Tal Iluz, Eran Gazit, Talia Herman, Eliot Sprecher, Marina Brozgol, Nir Giladi, Anat Mirelman, Jeffrey M Hausdorff

**Affiliations:** 1 Laboratory for Gait &Neurodynamics, Movement Disorders Unit, Department of Neurology, Tel Aviv Sourasky Medical Center, 6 Weizman Street, Tel Aviv 64239, Israel; 2 Sagol School of Neuroscience, Tel Aviv University, Tel Aviv, Israel; 3 Department of Physical Therapy, Tel Aviv University, Tel Aviv, Israel; 4 Department of Neurology, Sackler Faculty of Medicine, Tel Aviv University, Tel Aviv, Israel; 5 Harvard Medical School, Boston, MA, USA

**Keywords:** Parkinson’s disease, Gait, Fall risk, Body-worn sensors, Monitoring, Accelerometers

## Abstract

**Background:**

Falls are a leading cause of morbidity and mortality among older adults and patients with neurological disease like Parkinson’s disease (PD). Self-report of missteps, also referred to as near falls, has been related to fall risk in patients with PD. We developed an objective tool for detecting missteps under real-world, daily life conditions to enhance the evaluation of fall risk and applied this new method to 3 day continuous recordings.

**Methods:**

40 patients with PD (mean age ± SD: 62.2 ± 10.0 yrs, disease duration: 5.3 ± 3.5 yrs) wore a small device that contained accelerometers and gyroscopes on the lower back while participating in a protocol designed to provoke missteps in the laboratory. Afterwards, the subjects wore the sensor for 3 days as they carried out their routine activities of daily living. An algorithm designed to automatically identify missteps was developed based on the laboratory data and was validated on the 3 days recordings.

**Results:**

In the laboratory, we recorded 29 missteps and more than 60 hours of data. When applied to this dataset, the algorithm achieved a 93.1% hit ratio and 98.6% specificity. When we applied this algorithm to the 3 days recordings, patients who reported two falls or more in the 6 months prior to the study (i.e., fallers) were significantly more likely to have a detected misstep during the 3 day recordings (p = 0.010) compared to the non-fallers.

**Conclusions:**

These findings suggest that this novel approach can be applied to detect missteps during daily life among patients with PD and will likely help in the longitudinal assessment of disease progression and fall risk.

## Background

Falls are a leading cause of morbidity and mortality among older adults and patients with neurological disease like Parkinson’s disease (PD) [1-4]. Falls have a tremendous impact on functional independence, quality of life, and healthcare economics [2,5,6]. Much effort has been devoted to the development and evaluation of optimal measures of fall risk, mostly within laboratory settings [7-9]. However, among patients with PD, performance in the clinic or laboratory setting may not fully capture the risk of falls. Patients with PD suffer from motor response fluctuations that transiently affect motor function and fall risk. Fall risk may also be altered due to anxiety of testing in a clinic or due to the reverse “white coat syndrome”, where patients may apply an extra effort to walk well in front of a clinician. Tests of fall risk that reflect actual, everyday performance as a patient carries out his or her routine activities of daily living in the community and home setting may be more sensitive and may more fully reflect fall risk [10]. In previous work, we described a method of assessing fall risk in the home setting based on the quality of the gait pattern [11]. Here, we extend that approach to focus on the detection of missteps during community ambulation.

Missteps, also referred to as near falls, have been defined as a loss of balance that would result in a fall if sufficient recovery mechanisms are not activated [12]. The amount of self-reported missteps has been related to fall risk in PD and other populations [1,13-16]. Missteps are usually more frequent than falls and may occur before a person begins to fall, enhancing the potential predictive value of missteps. Unfortunately, self-report is, to a large degree, the gold-standard method for characterizing and quantifying missteps [17-19]. Moreover, self-report of missteps is limited by the subjective nature of recall bias and the long observation period that may be required for many missteps to be reported (e.g., weeks or months) [17,19].

The goal of the present study is to address the issue of objective, automated detection of missteps under real-world, daily life conditions in patients with PD. In the process of the algorithm development, we evaluated the ability of the algorithm to detect missteps in the laboratory setting during usual walking and during obstacle negotiation, designed to mimic missteps in real-life. To further validate the algorithm, we applied it to real-world data and compared the results between PD patients who reported falls and those who did not. We hypothesized that missteps would be more common in the PD fallers compared to PD non-fallers.

## Methods

### Subjects

40 PD patients participated in this study (subject characteristics and disease severity can be found in Table 1). Subjects were excluded if they had brain surgery in the past including deep brain stimulation implant or had significant co-morbidities likely to affect gait, e.g., acute illness, orthopedic disease, or history of stroke as well as subjects who could not walk independently during the off medication cycle period. A subject was classified as a faller if he/she reported at least two falls in the last six months. All subjects provide informed written consent before participating in this study. The protocol was approved by the local human studies committee.

**Table 1:** Subject characteristics (n = 40)

**Variables**	**Mean ± STD**	**Range**
Age [Yrs]	62.16 ± 10.02	41-81
Gender [f/m]	8/32	
Disease duration [Yrs]	5.34 ± 3.53	1-14.5
UPDRS at off	59.18 ± 21.96	29-108
Hoehn & Yahr	2.54 ± 0.66	2-4
Pull test	1.21 ± 1.29	0-3
Timed Up and Go [sec]	9.46 ± 2.46	5.63-17.79
Dynamic Gait Index	22.19 ± 1.81	16-24
Berg Balance Scale	53.12 ± 4.15	39 -56
Four Square Step Test	11.76 ± 3.12	7.45-19.5
Gait speed at off [m/sec]	1.15 ± 0.19	0.51-1.56
Number of fallers	9	
Mini Mental State Exam	29.18 ± 1.21	25-30

### Laboratory protocol

Subjects wore a small device (DynaPort Hybrid, McRoberts, The Hague, Netherlands; 87 × 45 × 14 mm, 74 g) that contained accelerometers and gyroscopes on the lower back, approximately at the level of L4-5. The triaxial accelerometer has a sensor range and resolution of ± 2 g and 0.001 g, respectively. The triaxial gyroscope has a sensor range and resolution of ± 100 deg/sec and 0.0069 deg/sec, respectively. Six channels were collected at 100 Hz each: vertical acceleration, medio-lateral acceleration, anterior posterior acceleration, and angular velocity in three directions: yaw, pitch and roll. Since missteps are relatively rare events, we designed a protocol to provoke missteps in the laboratory setting, aiming to mimic real-life missteps. Subjects walked in the following six conditions, each for approximately one to two minutes: 1) Comfortable, self-selected speed. 2) Comfortable speed while wearing a safety harness. 3) Fast walking. 4) While performing serial three subtractions (S3). 5) While negotiating obstacles. 6) While negotiating obstacles and subtracting serial 3s (i.e., the most challenging condition). The obstacles that the subjects negotiated were six sand like rubber plates (Terrasensa® HÜBNER GmbH) and 6 wooden sticks 1*60*10 cm covered in carpet placed in the subject’s path, and one transparent wire stretched at 10 cm high.

All walking besides the first condition were carried out while the subjects wore a safety harness that prevented actual falls. Any missteps were annotated in real-time by two clinicians. All sessions were videotaped to both validate the missteps and to ensure the detection of any misstep that may not have been observed in real-time. To use the annotated notes of the missteps, it was required to synchronize between the annotation and the data collected from the body-worn accelerometers. For that purpose, a stopwatch was activated at the same time that the sensors were turned on and was used by the clinician for the real-time annotations. Of note, the duration of a misstep typically ranges between half a second to several seconds. Thus, the precision required from the annotation and their synchronization is relatively high. To achieve such precision, every annotated misstep was observed in the videotape using highly accurate video editing software.

To diversify the data and enrich it for better performance during real-world testing, the acceleration sensor was also worn by the participants while they conducted functional performance-based assessments. As part of the protocol, each participant was assessed by a clinician using the following tools: Dynamic Gait Index [8], the Berg Balance Scale [20], the Timed Up and Go test [7] and the Four Square Step Test [21]. The Mini Mental State Examination [22] assessed global cognitive function. The acceleration data collected during these tests allowed us to check the algorithm on data that contain different situations resembling every day activity. Due to problems with the synchronization of the acceleration data with the video, only 33 subjects were included in the analysis used to develop the algorithm; all 40 subjects were included in the validation stage.

### Three day protocol

After the experiment in the laboratory was completed, the subjects wore the sensor for three consecutive days. They were asked to wear it during the day while they performed their daily normal routine. Subjects removed the device for sleeping and showering, replacing it themselves afterwards, as they practiced previously in the lab.

### Algorithm development

A variety of approaches to detect missteps were investigated. We derived an array of measures based on running time window on the acceleration including, for example, average, range, standard deviation, skewness, kurtosis, maximum and median amplitude value of each one of the six channels, and their derivatives. The signal magnitude and the normalized signal magnitude areas were calculated as well for two and three channels at a time. Other frequency-domain measures were based on the shape of the power spectral density: frequency, amplitude, width and slope of the dominant harmony of the power spectral density in the locomotion band (0.5-3.0 Hz) of all six channels. In addition, a complimentary approach was studied using wavelet analysis to identify localized changes in frequency characteristics of the signal. With all of these features, we tried several learning algorithms such as K-means [23], principal component analyses [24], and several types of machine learning (e.g., ‘Ada boost’, ‘Tree Bagger’ and ‘Bug’ [25]). The results from those algorithms were not satisfactory, as there were too many missteps that were not correctly detected. For example, ‘Ada boost’ with 20 trees detected only 62.5% of the missteps.

**Figure 1:**
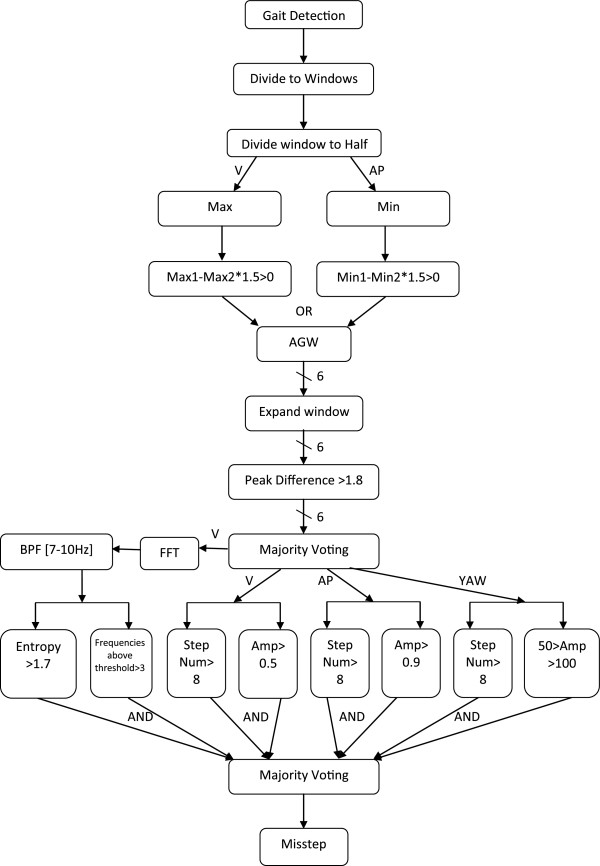
**The algorithm flow chart.** As shown, some of the steps of the algorithm are carried out sequentially and others in parallel. In the last step, a “majority rule” of the different channels is applied to determine if a given window is designated as a suspected misstep. Max - Maximum acceleration amplitude. Min - Minimum acceleration amplitude. Max1 - Acceleration amplitude of the highest peak. Max2 - Acceleration amplitude of the second peak. Min1- Acceleration amplitude of the lowest peak. Min2- Acceleration amplitude of the second lowest peak. AGW- Abnormal gait window. Amp-Amplitude.

We therefore developed a novel algorithm based on new features that were especially designed for the signal pattern observed during missteps in PD. Missteps, by definition, can only occur while walking or during gait initiation so the first stage was the extraction of gait segments. Good gait detection can substantially reduce false alarms generated by noise. During data collection in the laboratory, manual annotations were gathered to assist in locating gait segments afterwards. To make the gait extraction process more automated for the real-world data, a gait detection algorithm was developed. This first stage and the second stage, the automated detection of missteps (see Figure 1), are described below.

### Gait detection algorithm

The acceleration signal has a repeatable pattern in frequencies between 0.5-3.0 Hz during normal walking. The signal may deviate from this repeatable pattern due to various reasons such as tremor, alterations in the gait pattern, walking over or around obstacles, and placement of the sensor. Therefore, to minimize false detection of gait, the signal is band passed filtered [0.5-3.0 Hz] in order to extract only the gait frequencies.

The next stage of the algorithm uses a running window of 5-seconds in length on the vertical and anterior posterior acceleration axes. The data from these windows were convolved with one cycle of a 2 Hz sinusoidal signal that represents a cycle of gait in the filtered data. The resultant signal enables detection of gait by searching for local maxima which represents one gait cycle. Only windows at which 2–15 steps were detected were considered as gait. This range was chosen since gait typically takes place in the range of 0.5-3.0 Hz where 0.5 Hz reflects a stride every two seconds and 3.0 Hz reflects 3 steps every second. A 5 second windows therefore can contain 2–15 gait cycles.

### Misstep detection

The algorithm is made up of two parts. The main idea of the first part is to identify irregularities in the gait pattern and mark them as “suspected areas”. The role of the second part is to extract features that are more characteristic of missteps and remove windows that are likely not missteps. Most of the thresholds described below were determined according to trial and error after close inspection of the data.

The processing of the data was performed by dividing the gait data into 5-second windows, each window processed individually. A normalization process is applied on each window by subtracting its mean due to the placement of the sensor on the subjects that may sometimes be tilted or shift slightly during the trials. After the normalization, each window is divided into two equal time segments. For the vertical axis, the maximum in each segment is calculated, resulting in two maxima values. If the highest maximum is greater than one and a half times the lower one, then the window is classified as an abnormal window. A similar process is performed on the anterior posterior signal but the local minima are calculated at each segment instead of the maxima. If a window is found to be abnormal in one or both axes, it is classified as Abnormal Gait Window (AGW).

To further investigate the AGW, a wider environment around the window is examined to determine that the irregularity happens only within the 5 second window and that the change in the gait is not due to obstacle negotiation, the start or end of walking, or other non-misstep events. This environment is an extension of the window by a quarter of a window width on each side. We then compared the highest peak of the window and the third highest peak, which is located after it, in each window. If the ratio between them is greater than one point eight, the window is designated as a suspicious window (SW) that might contain a misstep. The idea is that the first peak is usually the impact at the beginning of the misstep and the third peak following it is already in the recovery stage or normal gait. If they are not different in their height, it is not a misstep. This process is preformed individually for each of the three acceleration axes and three gyroscope axes following their 0.5-20.0 Hz band pass filtering (to reduce noise). The decisions from the six channels enter a majority rule, which states that if more than three channels declare the window a SW, then it is labeled as such.

The second part of the algorithm is based on extracting features from only three channels: vertical, anterior posterior, and yaw. These axes contain the most relevant information to missteps. From each channel, we extract the maximum amplitude and the number of steps, calculated according to the number of peaks in the data. The requirements at each channel for an AGW to be a misstep are more than 8 steps per window and maximum window amplitude greater than 0.5 g for vertical, 0.9 g for anterior posterior, and between 50 deg/sec to 100 deg/sec for yaw. Two additional features are extracted from the FFT (Fast Fourier Transform) of the vertical axes after it is applied with a band pass filter between 7–10 Hz. The first one is entropy and the second one is the number of frequencies in the FFT signal with energy greater than 0.015 g. If the entropy is greater than one point seven and at least three frequencies above the aforementioned threshold, the window is declared as a misstep. Finally, if two or more channels (i.e., vertical, anterior posterior, yaw and FFT of the vertical axes) classify a window as a misstep, then it is labeled as such. A flow chart of the algorithm is presented in Figure 1.

### Algorithm assessment

#### Laboratory settings

To evaluate the algorithm, we used specificity and a “Hit ratio”, a measure that represents the number of detected missteps divided by the total number of missteps, instead of sensitivity. Whereas sensitivity requires detection of all the windows in which the missteps occurs, for a hit, it is sufficient that the event is detected at least in one window, even if it is spread over several windows.

#### Three day recordings

We tested the algorithm that was developed for the laboratory on the data from the 3 day recordings to determine if it can identify missteps that the subjects experience during ambulation at home and in the community. When applied to the 3 days data, we cannot ensure that the detections are indeed missteps, due to the lack of annotations, so it is only possible to label a window as a “suspected misstep” (sMS). The expectation was that the number of detections per subject will be higher for the fallers than for the non-fallers. When running the algorithm on the 3 days data, we normalized the number of detections (sMS) by the total walking time to compare between subjects, since the amount of walking for each subject varied greatly. The normalization was performed as follows:

$$\begin{array}{l} \mathrm{Normalized}\;\mathrm{sMS}=\left(\mathrm{Number}\;\mathrm{of}\;\mathrm{sMS}\;\mathrm{Windows}\right)\\ \;\left(/\mathrm{Number}\;\mathrm{of}\;\mathrm{Gait}\;\mathrm{Windows}\right)\\ \;\times 100 \end{array}$$

### Statistical analysis

Statistical analyses were conducted to provide indirect validation of the misstep detection model in patients with PD and to examine whether missteps detected by this algorithm, under real world conditions, differ between fallers and non-fallers. We hypothesized that PD fallers would have more missteps. Thus, a comparison of the likelihood of event detection between fallers to non-fallers, without direct knowledge of a true misstep, provides indirect validation of the detection method. Event/trial logistic regression models were employed, using SAS (SAS Institute, Cary, NC) PROC GENMOD, with laboratory condition detections as events, gait intervals as trials, fall history as an independent variable, and subjects as repeated factors assuming unstructured correlations.

## Results and discussion

### Laboratory settings

**Figure 2:**
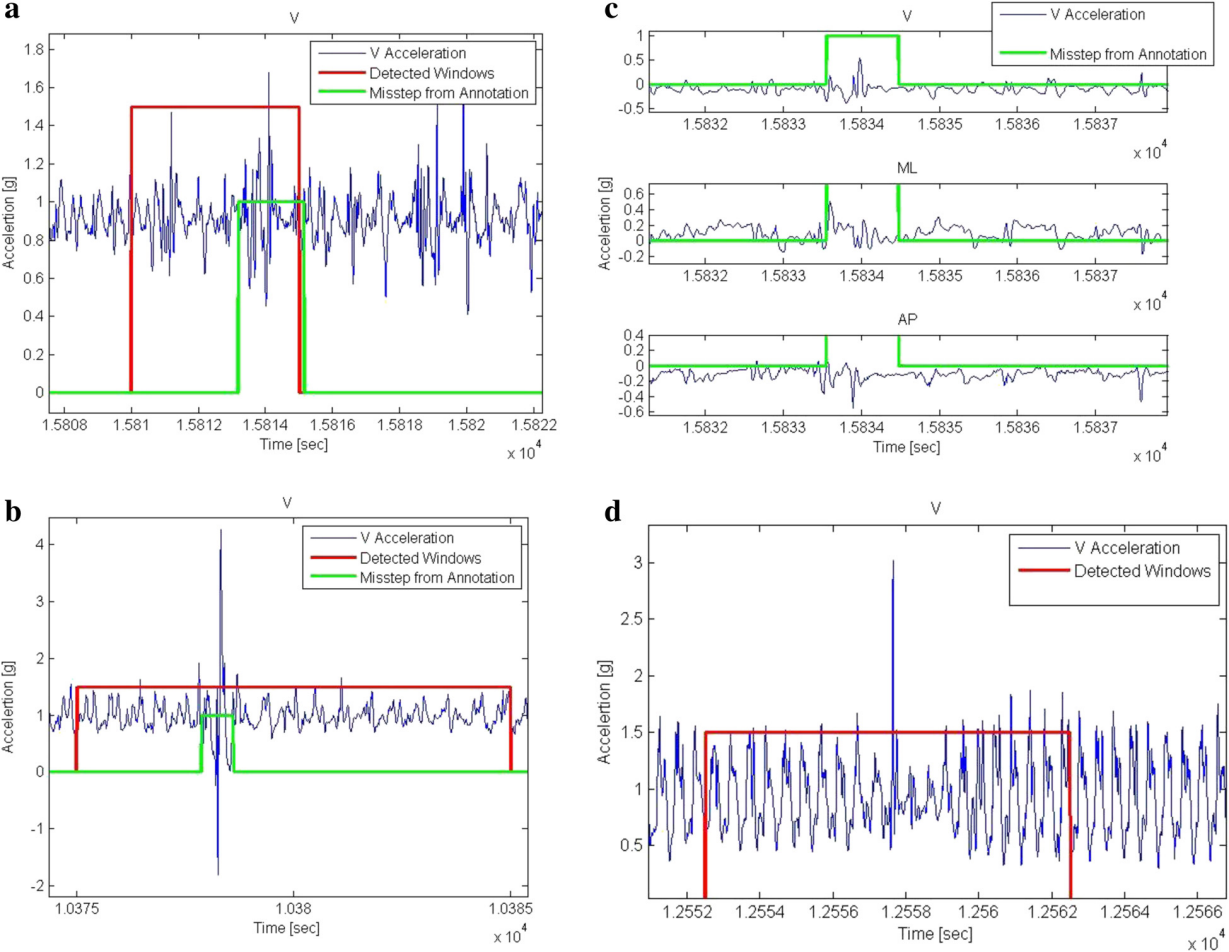
**Examples of correctly and incorrectly identified missteps. a)** Successful detection of a misstep occurring in the laboratory in the vertical acceleration. This misstep has relatively ***low*** acceleration, nonetheless, the change in the gait pattern is clear. **b)** Successful detection of a misstep occurring in the laboratory in the vertical acceleration. This misstep has relatively ***high*** acceleration with a clear change in the gait pattern. **c)** An example of a missed misstep. This event was not detected by the algorithm due to the low accelerations, and because the changes in the gait pattern are clear only in the vertical acceleration but not in the medio-lateral and anterior posterior directions. **d)** An example of a false alarm. In this gait window, there is very high amplitude and a clear change in the gait pattern due to obstacle negotiation, and therefore was not annotated as a misstep.

We recorded data for more than 60 hours, containing 29 missteps that occurred during the protocol from the 33 patients with PD. Most of the missteps occurred during the last two walking conditions when the subject walked while negotiating obstacles. Running the algorithm on the laboratory data achieved a 93.1% hit ratio and 98.6% specificity. False detections are changes in the acceleration signal that resemble a misstep in one or more key characteristics. Ultimately, however, they are not defined as a misstep (please recall Figure 1). Figure 2a and 2b depict two examples of detected missteps. In those missteps, like most other missteps, there are obvious changes in the acceleration pattern, however, there was a significant difference between the amplitudes in the two examples, as shown. The amplitudes of the missteps that we recorded were between 0.4 g to 6.0 g. (mean ± SD: 2.49 ± 1.3 g) Table 2 summarizes the values of key features of missteps and false alarms.

**Table 2:** Features extracted from the laboratory data

**Feature**	**Axis**	**Hit**	**Specificity**	**Misstep**		**False alarm**	
				**Mean**	**Std**	**Mean**	**Std**
Peak difference	Vertical [g]	93.10	83.31	0.34	0.18	0.60	0.20
	Medio-lateral [g]	89.65	81.38	0.33	0.17	0.59	0.19
	Anterior- posterior [g]	93.10	81.86	0.32	0.15	0.57	0.21
	Yaw [deg/sec]	86.20	83.27	0.46	0.14	0.66	0.18
	Pitch [deg/sec]	82.75	83.29	0.43	0.17	0.66	0.18
	Roll [deg/sec]	89.65	82.81	0.53	0.21	0.66	0.20
Frequencies above threshold	FFT- Vertical	86.20	89.10	8.08	4.57	5.20	3.39
Entropy				2.43	0.42	2.09	0.57
Number of steps	Vertical	48.27	93.58	10.76	1.73	10.18	1.77
Maximum amplitude [g]				1.86	1.06	0.97	0.59
Number of steps	Anterior- posterior	86.20	91.77	8.68	1.86	8.85	1.91
Maximum amplitude [g]				1.89	1.07	0.85	0.64
Number of steps	Yaw	55.17	95.89	8.08	4.57	5.20	3.39
Maximum amplitude [deg/sec]				2.43	0.42	2.09	0.57

The Hit and specificity for the first 6 features represent the performance of each feature on its own. For the other features, the results are the performance of each feature on its own but after the majority voting of the first 6.

Despite the benefits of pattern based features, there were many kinds of missteps and not all of them were suited to the patterns or only suited in one channel, while the algorithm was based on majority voting. An example of this problem can be seen in Figure 2c. Here there is a pattern of a misstep in the vertical axis, but there is not a significant pattern change in the medio-lateral or anterior posterior axes. The final goal was to develop an algorithm that would work on 3 days recordings from patients at home, therefore one of the main objectives was to reduce the number of false alarms to a minimum, even at the cost of not detecting all the missteps. Figure 2d shows an example of a false alarm that resembles a misstep both in the pattern and the amplitude. Some of those false alarms were reduced by changing the threshold, but not all of them.

### Three day recordings

**Figure 3:**
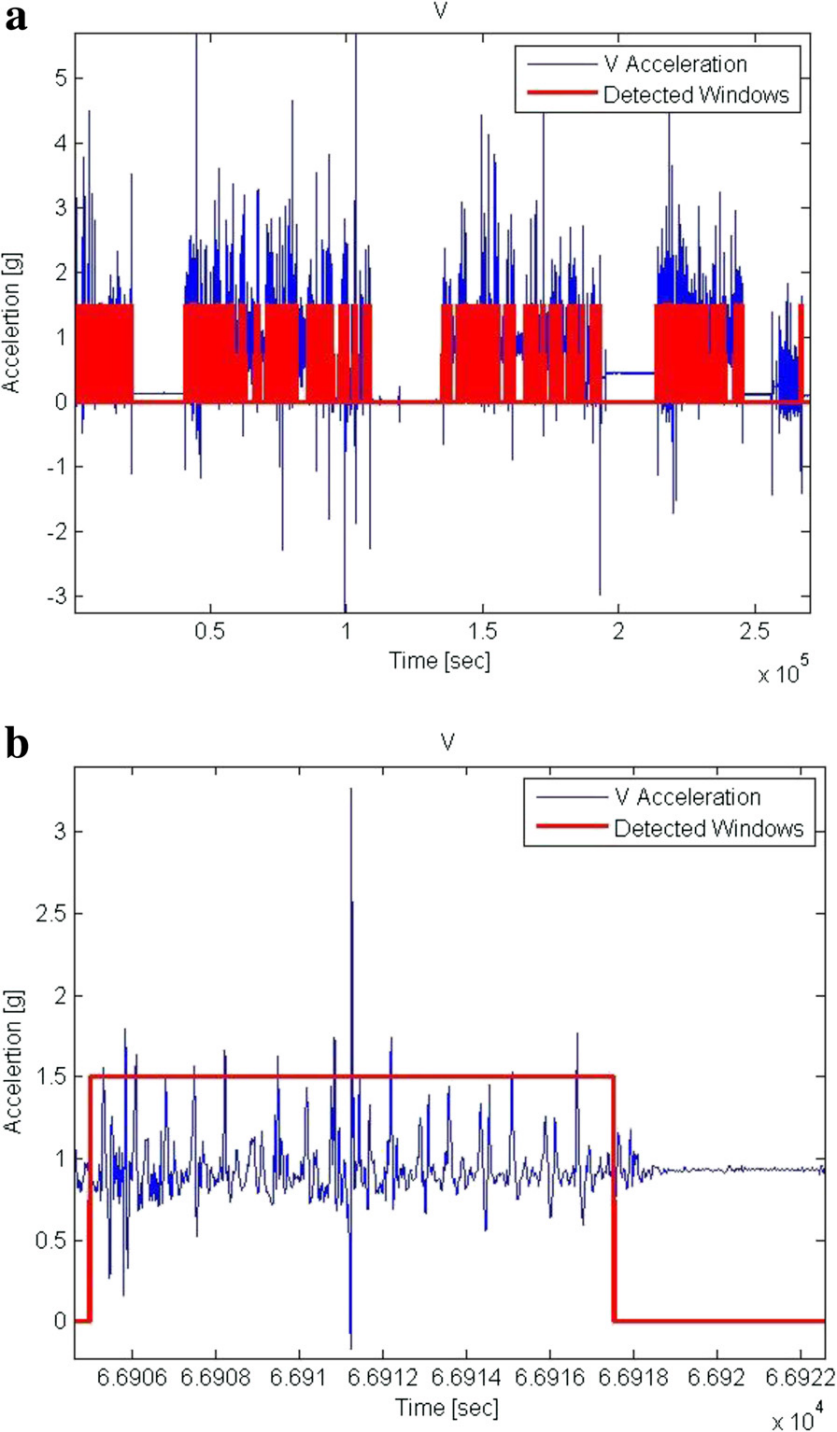
**Data from 3 days recordings. a)** Example of the entire data. **b)** Example of detected suspected missteps (sMS).

The results from the 3 days recordings were very diverse. Some subjects had a high number of sMS detections and some had only few. The same diversity was also observed in the number of gait windows. For example, the highest number of sMS was 1007 with 4148 gait windows (~5.7 hours of gait), while the lowest number of sMS was four with 95 gait windows (0.13 hours of gait). No correlation was found between the number of gait windows and the number of sMS. The high number of sMS could be explained using Figure 3a. It depicts gait of a faller and contains abnormalities in a large amount of gait windows, expressed both as high amplitudes and the inconsistent walk. The mean range of basic gait (in the laboratory) was 1.32 ± 0.3 g (vertical amplitude) while the mean gait range at home was 1.18 ± 0.65 g. To note that the range reached up to 9.2 g at home. In fact, 9.1% of the gait windows were at ranges above 2 g. Whereas some of the windows might actually be missteps, many are detected falsely as missteps, leading to a high number of sMS. Table 3 summarizes the features values for the sMS windows compare to gait windows.

**Table 3:** Features extracted from the 3 days recordings

**Feature**	**Axis**	**Suspected misstep**		**Not suspected misstep**	
		**Mean**	**Std**	**Mean**	**Std**
Peak difference	Vertical [g]	0.59	0.23	0.57	0.23
	Medio-lateral [g]	0.60	0.20	0.60	0.20
	Anterior- posterior [g]	0.56	0.20	0.55	0.23
	Yaw [deg/sec]	0.68	0.18	0.66	0.19
	Pitch [deg/sec]	0.64	0.21	0.63	0.20
	Roll [deg/sec]	0.65	0.20	0.64	0.20
Frequencies above threshold	FFT- Vertical	6.42	3.17	1.50	0.93
Entropy		2.46	0.34	1.39	0.45
Number of steps	Vertical	10.58	1.47	8.10	3.08
Maximum amplitude [g]		0.98	0.56	0.54	0.43
Number of steps	Anterior- posterior	9.94	0.99	6.84	2.81
Maximum amplitude [g]		1.34	0.5	0.43	0.35
Number of steps	Yaw	4.58	3.3	4.5	3.58
Maximum amplitude [deg/sec]		1.99	0.54	1.57	0.62

The division between sMS or not for each feature is according to the threshold of the feature.

Figure 3b depicts an example of sMS that seem to have all the features of a real misstep and it indeed was declared as one by the algorithm. Many of the detections resembled this one. Still, we do not claim that all the sMS are indeed missteps. Nonetheless, having experienced a fall in the prior 6 months resulted in an increased likelihood of producing detected sMS events in the 3 days recordings (P = 0.010). The odds ratio was 1.84 (95% confidence intervals: 1.15 – 2.93) that a faller would have a detected misstep during the 3 days recording, compared to a non-faller. The association between fall history and sMS tended to persist (p = 0.059) even after adjusting for age and disease duration. These findings provide a second level of indirect validation of the system: PD fallers tend to produce more misstep detections than non-fallers in the home and community setting.

Automatic fall risk assessment in the home environment has several potential advantages compared to a routine clinical assessment. Patient behavior in the clinic can be very different from that of everyday functioning for a variety of reasons [3,10]. “White coat syndrome”, motivation, the reverse white coat syndrome, and motor response fluctuations may all affect motor performance in the clinical setting; this is true in general, but is especially true among patients with PD. Thus, any onetime observation made in the clinic or laboratory must be taken with caution. These issues may be especially true when dealing with relatively rare events like falls or missteps. Since missteps occur infrequently and almost never in front of a clinician or in the lab, a method that can automatically detect these events and quantify their frequency should help to monitor and better evaluate fall risk, augmenting traditional assessment tools.

The current means of monitoring misstep frequency is self-report [13,14,17,18] Key advantages of self-report include low cost and simplicity. However, the costs of self-reporting are non-zero, as falls calendars, telephone calls and personnel time are typically used to maximize the reliability of this subjective process [26]. As sensor technology continues to improve and sensors become smaller and more ubiquitous, the costs should continue to drop. A fully re-usable, matchbox size, three axis body-fixed sensor that can be worn continuously for seven days and is only about 10% of the size of the device used in the present study can now be purchased for less than 100 euro (of course, each specific device has its advantages and disadvantages). When spreading the purchase costs over multiple testing, the price approaches that of self-report. While this configuration does not yet include a gyroscope for measuring angular velocity, no doubt in the near future this feature will also be added so that the algorithm described here can be fully applied using a low cost solution. Further studies comparing self-report and sensor based observations are required to fully establish the advantages and disadvantages of each approach. Nonetheless, the a priori advantages of objectivity and sensitivity argue in favor of using a sensor to assess missteps and suggest that perhaps they will be used to replace or augment self-report in the future. This possibility is consistent with the recent push for eHealth and mHealth where mobile technologies (e.g., smartphones, PDAs) are used to provide improved health services [27,28].

The present study is the first attempt at automatically detecting missteps in patients with PD or other patients with neurological impairment using a wearable sensor both in the laboratory setting and, critically, in the home environment. In the laboratory setting, the developed algorithm successfully identified misstep events with a good hit ratio and specificity. A previous pilot study in young and older adults also achieved good results in the laboratory setting [19]. Here we improved on the previous approach by using features that were especially designed to identify the pattern of missteps in patients with PD. The good detection rates in the laboratory setting, where each misstep is carefully annotated, is a key step and supports the validity of the developed algorithm. When applied to the 3 days recording, misstep detections were associated with fall status. This finding further supports the validity of the algorithm. Additionally, this finding is consistent with the assumption that missteps are related to fall risk [18,29]. The proposed algorithm is an important step towards automated monitoring of missteps among patients with PD.

A number of questions need to be addressed further. Three days appears to be sufficient to distinguish between fallers and non-fallers with respect to detected missteps, however, perhaps a longer recording period may provide more robust measures. For other applications, 7 days is recommended [30]. The nature of the collected data also creates some difficulties. Missteps provoked in the laboratory are not necessarily similar to missteps that occur at home or during community ambulation. In order to induce missteps in the laboratory, we used somewhat artificial conditions. These conditions may have resulted in missteps with relatively high amplitudes of accelerations and angular velocities. While observing the few missteps in the lab that occurred spontaneously, we saw that some of them have different characteristics. In future research with a larger sample size, it will be interesting to classify the different types of missteps according to amplitudes, temporal characteristics, patterns and other parameters and to study whether they are associated with certain broad categories of PD subjects and fall risk. The problem is that these kinds of “natural” missteps are much harder to create in the laboratory. There is no way to be 100% certain that any single one detection made in the 3 days recording is indeed a misstep. As shown in Figure 3a and 3b, it is difficult to visually differentiate between a false alarm and a real misstep due to the similarity in shape and features and due to the large amount of events. Nevertheless, the good results in the laboratory environment and the connection between the algorithm detections to fall risk in the home setting indicate that the majority of suspected missteps were likely to be missteps. Moreover, in contrast to the detection of falls, where each one is critical, here it may be less critical to achieve 100% accuracy and sensitivity.

One way of potentially improving detection accuracy is to add more sensors. However, this comes with a price as the current configuration is very simple and easy to apply. Larger scale prospective studies, perhaps with a longer observation periods are needed to further address these issues and to evaluate the potential of applying automated misstep detection using body-fixed sensors for the prospective monitoring of fall risk and motor function in patients with PD and others with neurological impairment.

## Conclusions

In this study, we developed a new algorithm for detecting missteps. When applying it to the home and community setting, PD fallers tend to produce about 23% more misstep detections compare to the non-fallers. These findings suggest that this novel approach can be applied to detect missteps during daily life among patients with PD. A larger scale study is needed to confirm the present results. Still, these initial results support the possibility that the described misstep detection algorithm will help in the longitudinal assessment of disease progression and fall risk.

## Competing interests

The authors declare that they have no competing interests.

## Authors’ contributions

TI design the algorithm, EG help to design the algorithm, TH conceived the design of the study, recorded the subject and performed the protocol, ES performed the statistical analysis, MB performed the protocol, NG, AM and JMH conceived the design of the study. All authors read and approved the final manuscript.
